# The Brooklyn Dental Society

**Published:** 1873-02

**Authors:** C. P. Crandell


### The Brooklyn Dental Society.

The fifth annual meeting of this society was held on Monday evening, October 7th, 1872. The President, Dr. A. H. Brockway, in the chair.

After the usual preliminary and general business, the association proceeded to the election of officers, with the following result. (See card)

The affairs of the society were shown to be in a most prosperous condition.

The Dental Infirmary so recently established bythe Society is no longer an experiment, but a pronounced success.

Much interest has been manifested in the clinics at the infirmary, many of which have been most instructive.

The thanks of the society were tendered to Dr. W. H. Atkinson, for the valuable services rendered by him in clinics the past year.

The meeting was one of interest and pleasure, followed by a sumptuous collation, provided by the retiring President.

C. P. Crandell, Rec. Sec.

